# Supplementary material for: LHX2 Enhances the Malignant Phenotype of Esophageal Squamous Cell Carcinoma by Upregulating the Expression of SERPINE2
Source: Genes (Basel). 2022 Aug 16;13(8):1457. doi: 10.3390/genes13081457 (PMC9408536; doi:10.3390/genes13081457)
Supplement: Supplementary file 1 [file genes-13-01457-s001.zip › genes-1848110-supplementary/Supplementary figures.pdf]

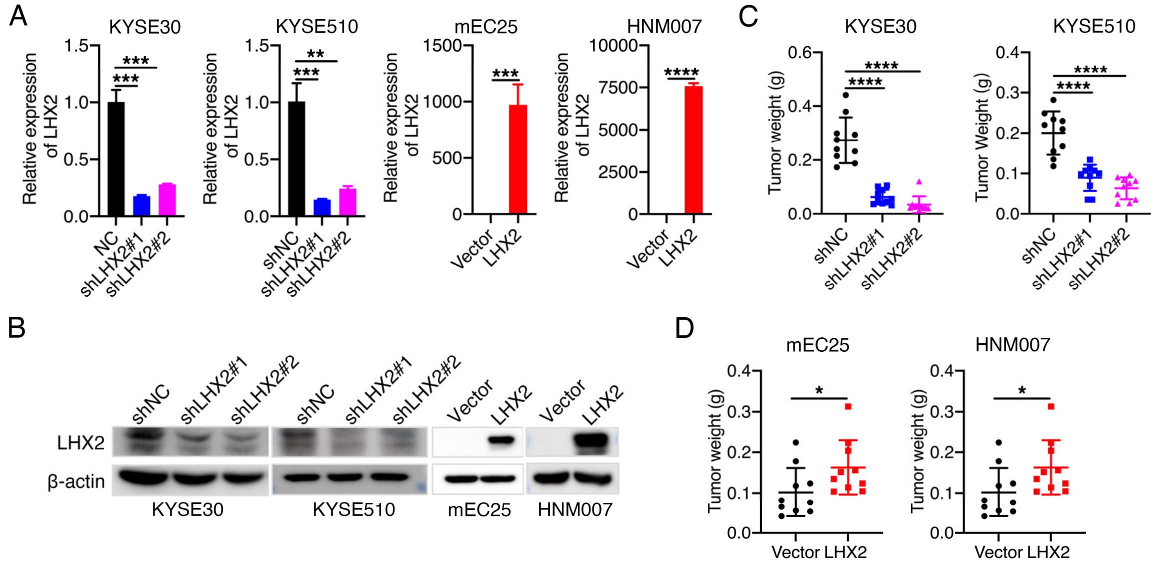

Figure S1. Knockdown of LHX2 inhibits ESCC tumor growth. The efficiency of LHX2 knockdown in KYSE30/KYSE510 cells, and LHX2 overexpression in mEC25/HNM007 cells was detected by qRT-PCR (**A**) and western bolting (**B**). Tumor weight of mice subcutaneously injected with LHX2-knockdown KYSE30/KYSE510 cells (**C**), LHX2-overexpressing mEC25/HNM007 cells (**D**) and control cells (n = 10). Data are presented as the mean ± SD, two-tailed t-tests, $^{**}P < 0.01$, $^{***}P < 0.001$, $^{****}P < 0.0001$.

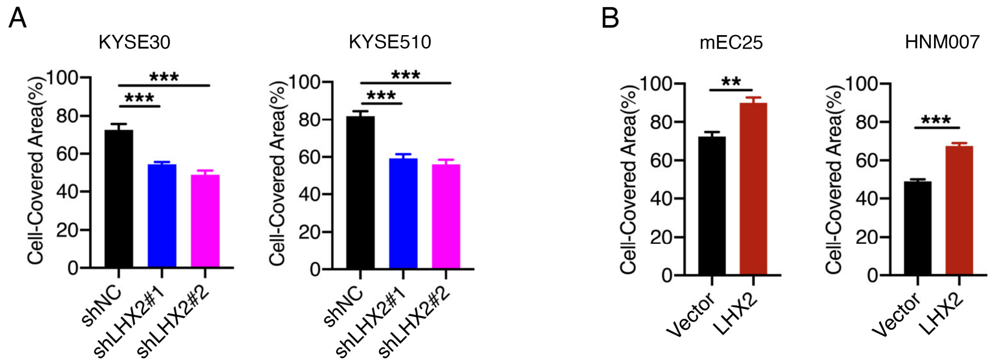

Figure S2. Knockdown of LHX2 inhibits the wound-healing of ESCC cells. The quantification of wound-healing assays of LHX2-knockdown KYSE30/KYSE510 cells (**A**), LHX2-overexpressing mEC25/HNM007 cells (**B**) and their corresponding control cells. $^{**}P < 0.01$, $^{***}P < 0.001$.

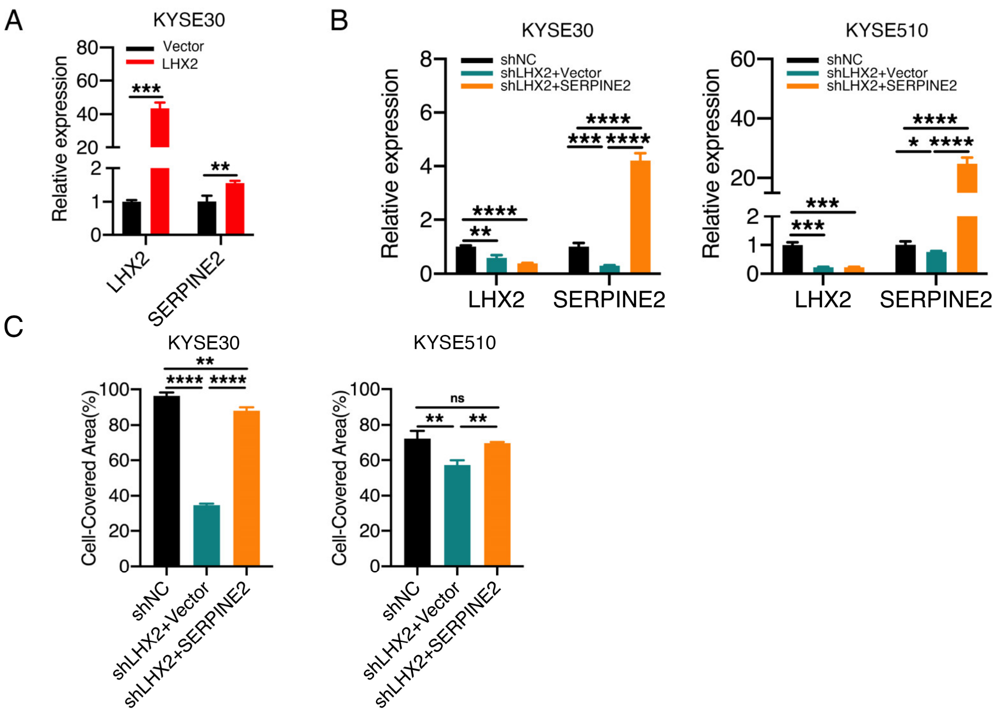

Figure S3. LHX2 transcriptionally regulates the expression of SERPINE2. (**A**) The efficiency of LHX2 and SERPINE2 was detected by qRT-PCR in KYSE30 cells transfected with pcDNA3-LHX2-Flag plasmids. (**B**) The expression of SERPINE2 was detected by qRT-PCR in LHX2-knockdown KYSE30/KYSE510 cells. (**C**) The quantification of wound-healing assays of exogenous overexpression of SERPINE2 on functional recovery in LHX2-knockdown KYSE30/KYSE510 cells and their control cells. Data are presented as the mean ± SD, two-tailed t-tests, **P* < 0.05, ***P* < 0.01, ****P* < 0.001, ****P < 0.0001. ns means no significance.
